# Enhanced Energy Storage Performance in La-Doped CaBi_4_Ti_4_O_15_ Films Through the Formation of a Weakly Coupled Relaxor

**DOI:** 10.3390/nano14241998

**Published:** 2024-12-13

**Authors:** Quanlong Liu, Lei Zhang, Jun Ouyang, Yan Liu, Zhehong Tang, Jieyu Chen, Fei Guo, Yunpeng Zhou

**Affiliations:** 1 College of Science, Inner Mongolia University of Technology, Hohhot 010051, China; 20010000037@imut.edu.cn (Q.L.); 20231100055@imut.edu.cn (L.Z.); 20241100061@imut.edu.cn (Y.L.); guofei@imut.edu.cn (F.G.); 2 Discharge Plasma and Functional Materials Application Laboratory, Inner Mongolia University of Technology, Hohhot 010051, China; 3 Institute of Advanced Energy Materials and Chemistry, School of Chemistry and Chemical Engineering, Qilu University of Technology (Shandong Academy of Sciences), Jinan 250353, China; ouyangjun@qlu.edu.cn

**Keywords:** energy storage, weakly coupled relaxor, breakdown strength, PNRs

## Abstract

Relaxor ferroelectric film capacitors exhibit high power density with ultra-fast charge and discharge rates, making them highly advantageous for consumer electronics and advanced pulse power supplies. The Aurivillius-phase bismuth layered ferroelectric films can effectively achieve a high breakdown electric field due to their unique insulating layer ((Bi_2_O_2_)^2+^ layer)). However, designing and fabricating Aurivillius-phase bismuth layer relaxor ferroelectric films with optimal energy storage characteristics is challenging due to their inherently stable ferroelectric properties. In this work, lead-free CaBi_4-*x*_La*_x_*Ti_4_O_15_ films were synthesized using the sol–gel technique and a weakly coupled relaxor design. On one hand, the introduction of La^3+^ ions weaken the dipole–dipole interactions, thereby enhancing the relaxor behavior. Alternatively, the expansion of grain size is restricted to enhance the number of grain boundaries, which possess improved insulating properties. This leads to a higher breakdown electric field. The results indicate that CaBi_4-*x*_La*_x_*Ti_4_O_15_ (*x* = 1.0) films exhibit excellent recoverable energy storage density (70 J/cm^3^) and high energy efficiency (73%). Moreover, the film exhibited good temperature stability and frequency stability. This study not only identifies a promising material for dielectric film capacitors but also demonstrates that the energy storage capabilities of Aurivillius-phase bismuth layer ferroelectric films can be effectively modulated through a design incorporating weakly coupled relaxor characteristics.

## 1. Introduction

With the continuous development of advanced electronics and power systems, energy storage technologies have gained increasing significance, especially in areas such as independent transportation, electronic communications, and medical facilities [1,2,3]. Therefore, it is very important to accelerate the research of electric energy storage technologies. Fuel cells, secondary batteries, supercapacitors, and dielectric capacitors have become the research subjects of various energy storage systems [4,5]. Among them, dielectric capacitors, which store electrical energy in the form of electric displacement, exhibit exceptional attractiveness due to their rapid charging and discharging rates, high power density, and substantial operating voltage [6,7,8]. However, the relatively low energy storage density poses challenges for its miniaturization, integration, and compactness in energy storage devices [8,9]. Enhancing the energy storage performance of dielectric capacitors is crucial in order to minimize the size and cost of advanced electronic and power equipment, thus necessitating a focus on optimizing their capabilities. In general, the recoverable energy density (*W_rec_*) and energy storage efficiency (*η*) of a dielectric material can be estimated by integrating its electric hysteresis loop, which can be calculated using the following formula [2,6,9]:

(1) $$W_{rec}=\int_{p_{r}}^{P_{\max}}EdP$$

(2) $$W=W_{rec}+W_{loss}$$

(3) $$\eta =\frac{W_{rec}}{W}=\frac{W_{rec}}{W_{rec}+W_{loss}}$$

where *P*_max_, *P*_r,_ and *E* denote the maximum polarization, residual polarization, and applied electric field in the hysteresis loop, respectively. It is evident that dielectric materials with high *E* and large polarization difference Δ*P* (i.e., Δ*P* = *P*_max_ − *P*_r_) may be potential candidates for energy storage applications. Here, *W* represents the total energy storage density, and *W*_loss_ denotes the energy loss resulting from hysteresis effects.

**Figure 1:**
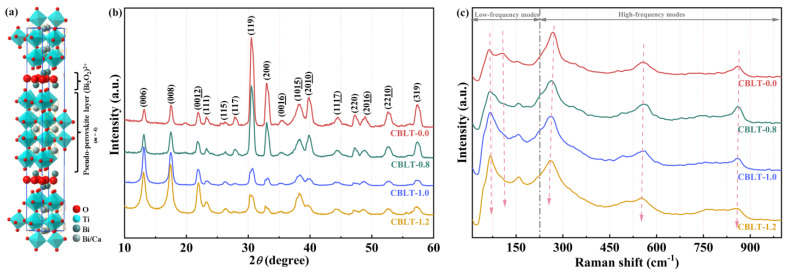
(**a**) The crystal structure diagram of CaBi_4_Ti_4_O_15_ film. (**b**) XRD patterns of CBLT-*x* (*x* = 0.0, 0.8, 1.0, and 1.2) films. (**c**) Raman spectra of CBLT-*x* (*x* = 0.0, 0.8, 1.0, and 1.2) films.

Relaxor ferroelectric (RFE) and antiferroelectric (AFE) materials have been extensively studied in the field of energy storage due to their high dielectric constants, which are associated with *P*_max_, and their moderate disruptive electric field compared to linear dielectrics [10,11,12]. For instance, Shu et al. [11] incorporated “isolated polar slush” into RFE films and prepared Bi(Mg_0.5_Ti_0.5_)O_3_-SrTiO_3_-based RFE films with a *W*_rec_ of up to 202 J/cm^3^ using the sol–gel method. Zhu et al. [12] achieved a breakdown electric field of up to 1450 kV·cm^−1^, *W*_rec_~14 J·cm^−3^, and *η*~85% in Sm_0.05_Ag_0.85_Nb_0.7_Ta_0.3_O_3_ multilayer capacitors by utilizing the displacement fluctuation strategy involving heterovalently doped atoms. However, the natural RFEs and AFEs exhibit limited diversity and predominantly contain lead, which imposes certain constraints on their applicability in the field of energy storage. Fortunately, it is feasible to optimize RFEs and AFEs within FEs [13,14,15]. Recently, there has been significant research interest in Bi-containing ferroelectric materials as promising alternatives to lead-based dielectrics due to the highly polarized properties of Bi^3+^ ions, which are similar to Pb^2+^ ions with 6s lone electron pairs [16,17]. The Aurivillius phase Bi layer-structured ferroelectrics (BLSFs) exhibit the characteristics of high ferroelectric polarization, good insulation properties, and excellent fatigue resistance. BLSFs materials were first proposed by B. Aurivillius in 1949 [18,19]. Their general formula is represented as (Bi_2_O_2_)^2+^(*A_m_*_-1_*B_m_*O_3*m*+1_)^2−^, where *A* represents univalent, bivalent, or trivalent ions (such as Na^+^, K^+^, Pb^2+^, Sr^2+^, Bi^3+^, etc.), allowing the formation of twelve-coordination structures; *B* denotes the transition metal element (such as Fe^3+^, Ti^4+^, Zr^4+^, Nb^5+^, W^6+^, etc.) that is suitable for forming oxygen octahedra. The value of *m* represents a positive integer, indicating the number of *B*O_6_ octahedrons in each (*A_m_*_-1_*B_m_*O_3*m*+1_)^2−^ layer [20,21,22,23]. By enabling an increase in the quantity of stacked perovskite structural units (*m*), a larger array of members can be generated. The (*A_m_*_-1_*B_m_*O_3*m*+1_)^2−^ layer and (Bi_2_O_2_)^2+^ layer of BLSFs grow alternately along the *c*-axis, forming a natural superlattice structure. Moreover, the (Bi_2_O_2_)^2+^ layers can accommodate multiple perovskite units, which has significant research implications. The CaBi_4_Ti_4_O_15_ (where *m* = 4) is an environmentally friendly ferroelectric material with a bismuth layer structure. Figure 1a depicts the schematic representation of the crystal structure of CaBi_4_Ti_4_O_15_ film. It can be observed that the A site in the (CaBi_2_Ti_4_O_13_)^2−^ layer exhibits a random distribution of Bi^3+^ and Ca^2+^. The B site is occupied by Ti^4+^, which are positioned at the center of the octahedron and exhibit strong ferroelectric properties. The key challenge in the field of energy storage for CaB_i4_Ti_4_O_15_ lies in effectively disrupting the macroscopic ferroelectric domains with strong interaction, reducing *P*_r_, and increasing *E*_b_. Numerous studies have demonstrated that the incorporation of rare-earth ions effectively induces relaxor behavior and enhances energy storage performance [24,25,26]. For example, (Ce, Mn)-modified (Na_0.8_K_0.2_)_0.5_Bi_0.5_TiO_3_-BiFeO_3_ film has a larger Δ*P* and achieved a *W*_rec_ of 58.3 J/cm^3^ and an efficiency of 58.1% under 2200 kV/cm [24]. Vu et al. [25] reported Sm-doped Ba(Zr_0.35_Ti_0.65_)O_3_ relaxor ferroelectric films with *W*_rec_ of 133.3 J/cm^3^ and *η* of 89.4%. In our recent work, SrBi_4_LaTi_4_FeO_18_ film achieved a high *W*_rec_ of 142.8 J/cm^3^ and an excellent *η* of 80% [26]. The outstanding outcomes demonstrate that incorporating rare-earth ions is an effective approach to improving the energy storage capabilities of ferroelectric materials.

In this work, the sol–gel technology was employed to achieve films of CaBi_4_Ti_4_O_15_ doped with rare-earth ions (La^3+^), aiming to investigate the impact of La^3+^ doping on the energy storage properties of CaBi_4_Ti_4_O_15_ film through the formation of a weakly coupled relaxor. On one hand, the substitution of Bi^3+^ with La^3+^ weakens the original ferroelectric domain structure due to the diminished orbital coupling between La-O bonds, thereby resulting in the formation of highly dynamic and sensitive polar nanoregions (PNRs) [27,28]. On the other hand, the valence states of La^3+^ and Bi^3+^ are identical, and their ionic radii coordinated by the XII coordination are both 1.36 Å. Substituting Bi^3+^ with La^3+^ can effectively reduce the concentration of oxygen vacancies in CaBi_4_Ti_4_O_15_ films [29]. All of these have the potential to enhance both the *W*_rec_ and *η* of CaBi_4_Ti_4_O_15_ film. As a result, the CaBi_3_LaTi_4_O_15_ film achieves a *W*_rec_ of up to 70 J/cm^3^ and an *η* of up to 73% under 2050 kV/cm while demonstrating excellent temperature stability (−20 °C~120 °C) and frequency stability (0.2 kHz~20 kHz). This is mainly due to the weakly coupled relaxor caused by La^3+^ doping. The subsequent discussion will comprehensively elucidate on the regulation of energy storage performance through weakly coupled relaxor induced by La^3+^ doping.

## 2. Experimental Section

### 2.1. Materials Preparation

The CaBi_4-*x*_La*_x_*Ti_4_O_15_ (CBLT-*x*, *x* = 0.0, 0.8, 1.0, and 1.2) films were fabricated on Pt/Ti/SiO_2_/Si (100) substrates by the sol–gel method. The primary experimental raw materials employed include hexahydrate lanthanum nitrate [La(NO_3_)_3_·6H_2_O (99.99%, Innochem)], pentahydrate bismuth nitrate [Bi(NO_3_)_3_·5H_2_O (99%, Innochem)], titanium tetrabutoxide [C_16_H_36_O_4_Ti (98+%, Innochem)], calcium acetate [C_4_H_6_O_4_Ca (99%, Innochem)], ethylene glycol monomethyl ether [C_3_H_8_O_2_ (99.5%, Innochem)], and acetylacetone [C_5_H_8_O_2_ (99%, Innochem)]. To prepare the CBLT-*x* precursor solutions, the quantities of La(NO_3_)_3_·6H_2_O, Bi(NO_3_)_3_·5H_2_O, C_4_H_6_O_4_Ca and C_16_H_36_O_4_Ti should be accurately weighed according their respective mole ratios in the chemical formula. Subsequently, the compounds are dissolved sequentially in C_3_H_8_O_2_ to obtain the precursor solutions of CBLT-*x*. To compensate for the volatilization of bismuth during the rapid annealing process, an 10% excess of Bi(NO_3_)_3_·5H_2_O was employed to account for the loss of bismuth, while a controlled quantity of C_5_H_8_O_2_ was introduced to ensure the stability of the solutions. After aging for 120 h, the CBLT-*x* precursor solutions were spin-coated onto Pt/Ti/SiO_2_/Si(100) substrates and placed on a preheated hot plate at 280 °C for 300 s. Following the baking process, the samples underwent oxygen ions plasma treatment for 180 s and then rapidly annealed in an oxygen atmosphere at 750 °C for 180 s. The aforementioned steps should be repeated eight times, and the ultimate rapid annealing at 750 °C for 900 s can successfully prepare the CBLT-*x* (*x* = 0.0, 0.8, 1.0, and 1.2) films.

### 2.2. Characterization of Materials

The crystalline structure of the CBLT-*x* (*x* = 0.0, 0.8, 1.0, and 1.2) films was analyzed using X-ray diffraction (XRD, Rigaku Smart Lab, Tokyo, Japan) and Raman spectroscopy (Thermo Fisher Scientific, DXR2 Xi, Waltham, MA, USA). The surface morphology, cross-sectional views, and elemental distribution of the films were examined using scanning electron microscopy (SEM, Hitachi SU-4800, Tokyo, Japan) and energy-dispersive spectroscopy (EDS, Hitachi SU-4800, Tokyo, Japan). The film’s domain structure was analyzed using piezoresponse force microscopy (PFM, Asylum Research Cypher, Long Beach, CA, USA). The valence states of the elements were identified through X-ray photoelectron spectroscopy (XPS, Thermo Fisher Scentific EscaLab 250Xi, Waltham, MA, USA). To facilitate the characterization of relevant electrical properties, a small-scale magnetron sputtering technique was employed to deposit circular gold top electrodes with a diameter of 0.2 mm onto the film’s surface. The dielectric constant and loss were measured as a function of temperature utilizing an impedance analyzer (HCT1821, Tongguo Tech., Dongguan, China) connected to a variable-temperature probe. The multiferroic testing system (MultiFerroic 500 V, Radiant, El Segundo, CA, USA) is used for testing the hysteresis loops and leakage current characteristics. The films’ direct energy storage capacities are measured using an electrical dielectric charging and discharging test system (CFD-003, Tongguo Tech., Dongguan, China).

## 3. Results and Discussion

The XRD patterns of CBLT-*x* (*x* = 0.0, 0.8, 1.0, and 1.2) films are presented in Figure 1a. It is evident that all the films exhibit a polycrystalline nature and can be characterized by the orthorhombic perovskite structure (space group *A*2_1_*am*) of BaBi_4_Ti_4_O_15_, as referenced in JCPDS Card No. 35-0757 [30,31]. The XRD pattern of the CBLT-0.0 film exhibits a random orientation, with the primary diffraction peak located at (119). The presence of a four-layer Aurivillius-phase structure in the CBLT-*x* (*x* = 0.0, 0.8, 1.0, and 1.2) films is confirmed by the observation that the highest diffraction peak (112*m*+1) aligns with previously reported data [32]. As the La^3+^ doping concentration increases, the intensity of the (119) peaks in the CBLT-*x* films gradually decreases. According to the Scherrer formula, a gradual increase in the half-width of the main peak (119) in the CBLT-*x* (*x* = 0.0, 0.8, 1.0, and 1.2) films indicates a concomitant reduction in grain size [33]. Reducing the grain size is beneficial for improving *E*_b_. The peak intensities of (006), (008), and (0012) peaks gradually increase with the concentration of La^3+^, indicating a pronounced preferential orientation along the *c*-axis for the CBLT-*x* (*x* = 0.0, 0.8, 1.0, and 1.2) films. Furthermore, the longer La-O bond length compared to the Bi-O bond length results in a shift of the (200) diffraction peak towards lower angles in the CBLT-*x* films, leading to an expansion of the lattice volume. This finding indicates the successful integration of La^3+^ into the lattice structure of CBLT-*x* (*x* = 0.0, 0.8, 1.0, and 1.2) films. The Raman scattering spectroscopy was utilized to examine the crystal structure of the CBLT-*x* (*x* = 0.0, 0.8, 1.0, and 1.2) films, as shown in Figure 1b. The low-frequency Raman spectra of the BLSFs are associated with the vibrational modes of Bi^3+^, while the high-frequency modes are linked to the distortion and stretching of the crystal lattice and the *B*O_6_ octahedron [34,35]. At the low-frequency region, the modes at ~62.9 cm^−1^ and ~155.8 cm^−1^ can be attributed to the presence of Bi^3+^ within the (Bi_2_O_2_)^2+^ layers. The observed shift towards higher frequencies for these modes (~62.9 cm^−1^ and ~155.8 cm^−1^) with increasing La^3+^ content suggests that La^3+^ has replaced Bi^3+^ in the (Bi_2_O_2_)^2+^ layers [36]. The mode at ~107.3 cm^−1^ is attributed to Bi^3+^ in pseudo-perovskite (CaBi_2_Ti_4_O_13_)^2−^ slabs. The observed diffusion and shift of this mode with increasing of La^3+^ concentration indicates the substitution of Bi^3+^ by La^3+^ in pseudo-perovskite (CaBi_2_Ti_4_O_13_)^2−^ slabs. In the high-frequency region, the peaks at approximately 268.6 cm^−1^ and 555.9 cm^−1^ can be attributed to the stretching and torsional modes exhibited by the TiO_6_ octahedra [35]. The two modes progressively transition towards the lower frequency range, indicating a high substitution rate of La^3+^ for Bi^3+^ in the CBLT-*x* (*x* = 0.0, 0.8, 1.0, and 1.2) films, thereby effectively stabilizing the TiO_6_ octahedra. This occurrence is mainly ascribed to the higher metallic nature of La in comparison with Bi [37,38]. This characteristic enables La to effectively reduce oxygen vacancies within the pseudo-perovskite (CaBi_2_Ti_4_O_13_)^2−^ layers, resulting in the stabilization of TiO_6_ octahedra and an improvement in *E*_b_. The mode at ~862.7 cm^−1^ is associated with the extension of TiO_6_ octahedral chains between the layers of (Bi_2_O_2_)^2+^. The alteration in this mode suggests that the substitution of La^3+^ within the (Bi_2_O_2_)^2+^ layers impacts the vibration of the TiO_6_ octahedra. This effect occurs through an interaction between the pseudo-perovskite (CaBi_2_Ti_4_O_13_)^2−^ layer and the (Bi_2_O_2_)^2+^ layer, thus reducing structural distortion. Combined with the results of XRD and Raman spectroscopy, it is evident that La^3+^ has successfully incorporated into the lattice of CBLT-*x* (*x* = 0.0, 0.8, 1.0, and 1.2) films, thereby enhancing the stability of TiO_6_ octahedra.

**Figure 2:**
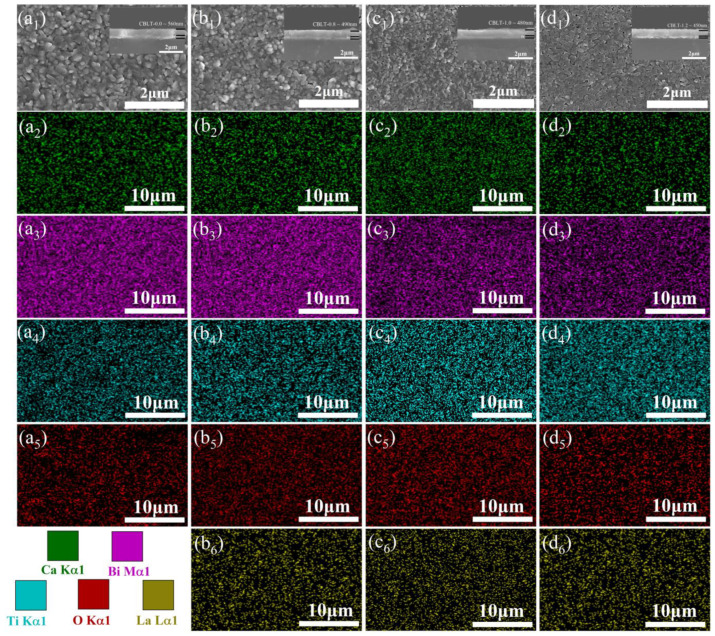
(**a_1_**–**d_1_**) Surface SEM images of CBLT-*x* (*x* = 0.0, 0.8, 1.0, and 1.2) films, and the inset shows the corresponding cross-sectional SEM images of CBLT-0.0, CBLT-0.8, CBLT-1.00, and CBLT-1.2 films, respectively. (**a_2_**–**a**_5_) EDS mapping images of specific elements (Ca, Bi, Ti, and O) of CBLT-0.0 film. (**b_2_**–**b**_6_) EDS mapping images of specific elements (Ca, Bi, La, Ti, and O) of CBLT-0.8 film. (**c_2_**–**c_6_**) EDS mapping images of specific elements (Ca, Bi, La, Ti, and O) of CBLT-1.0 film. (**d_2_**–**d_6_**) EDS mapping images of specific elements (Ca, Bi, La, Ti, and O) of CBLT-1.2 film.

The surface of CBLT-0.0, CBLT-0.8, CBLT-1.00, and CBLT-1.2 films can be visualized in Figure 2(a_1_–d_1_) using SEM. All CBLT-*x* (*x* = 0.0, 0.8, 1.0, and 1.2) films exhibit crack-free, dense, and smooth microstructural surface. It appears that, as the concentration of La^3+^ doping increases, there is a reduction in the size of grains within CBLT-*x* (*x* = 0.0, 0.8, 1.0, and 1.2) films while simultaneously increasing the number of grain boundaries. Meanwhile, a significant decrease in the number of pores has been observed in the CBLT-*x* (*x* = 0.0, 0.8, 1.0, and 1.2) films. The primary cause of this phenomenon is the control of grain growth by the migration rate at grain boundaries, which depends on the concentration of vacancy lattice points [39]. Substituting La^3+^ for Bi^3+^ effectively reduces the concentration of oxygen vacancies and slows down the diffusion rate of large grain growth. This process facilitates the enhancement of the *E*_b_ in CBLT-*x* (*x* = 0.0, 0.8, 1.0, and 1.2) films. The EDS elemental distribution maps of the CBLT-0.0 film are presented in Figure 2(a_2_–a_5_), while Figure 2(b_2_–b_6_) illustrates the EDS elemental distribution maps of the CBLT-0.8 film. Additionally, Figure 2(c_2_–c_6_) displays the EDS elemental distribution maps of the CBLT-1.0 film, and Figure 2(d_2_–d_6_) showcases the EDS elemental distribution maps of the CBLT-1.2 film. The specific elements (Ca, Bi, La, Ti, and O) are found to be distributed uniformly across all the films. The illustrations in Figure 2(a_1_–d_1_) show cross-sectional images of the corresponding films. It can be observed that all CBLT-*x* (*x* = 0.0, 0.8, 1.0, and 1.2) films exhibit a uniform and dense interfacial structure. The thicknesses of CBLT-0.0, CBLT-0.8, CBLT-1.0, and CBLT-1.2 films are approximately 560, 490, 480, and 450 nm, respectively.

**Figure 3:**
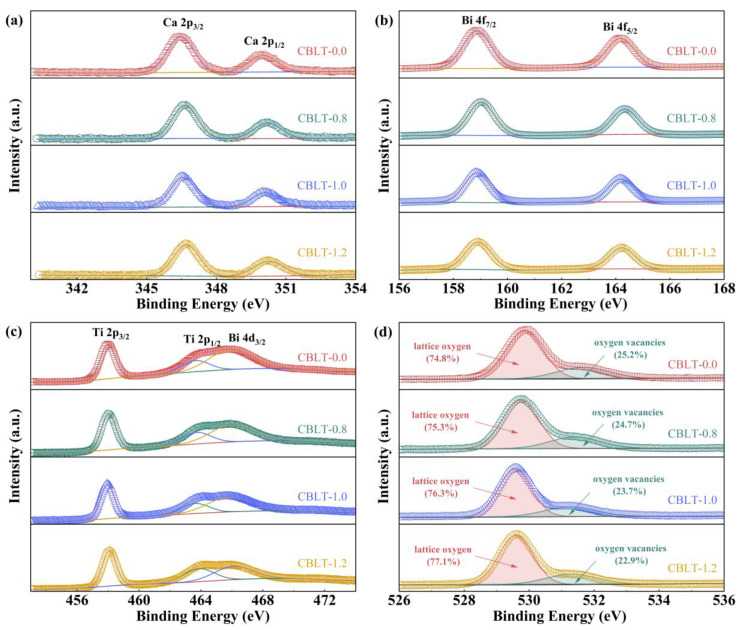
(**a**) Ca 2p, (**b**) Bi 4f, (**c**) Ti 2p and (**d**) O 1s XPS spectra of CBLT-*x* (*x* = 0.0, 0.8, 1.0, and 1.2) films.

XPS spectrum was employed as a surface-sensitive chemical analysis technique to investigate the valence states of Ca, Bi, Ti, and O elements in CBLT-*x* (*x* = 0.0, 0.8, 1.0, and 1.2) films. To minimize the influence of oxygen adsorption on XPS findings for the CBLT-*x* (*x* = 0.0, 0.8, 1.0, and 1.2) films, an in situ Ar ion beam etching procedure was performed on the pristine films under high vacuum conditions. Subsequently, the precision of analyzing oxygen vacancies was improved through the implementation of in situ XPS characterization. Figure 3a–c show the XPS spectra of Ca 2p, Bi 4f, and Ti 2p, respectively. The binding energies of Ca 2p_1/2_ and Ca 2p_3/2_ exhibit a slight shift, with values changing from 349.99 eV to 350.29 eV and from 346.45 eV to 346.65 eV, respectively. Similarly, the peak positions for Bi 4f_5/2_ and Bi 4f_7/2_ experience a minor displacement, transitioning from 164.19 eV to 164.23 eV and from 158.83 eV to 158.94 eV, respectively. Additionally, the binding energies of Ti 2p_1/2_ and Ti 2p_3/2_ also demonstrate a slight alteration, shifting slightly from 463.85 eV to 463.88 eV and from 457.98 eV to 458.10 eV, respectively. The results indicate that the binding energy of Ca 2p, Bi 4f, and Ti 2p increases as the La^3+^ doping concentration rises. This implies that the creation of oxygen deficiencies is impeded [40]. The O 1 s XPS spectra of the CBLT-*x* (*x* = 0.0, 0.8, 1.0, and 1.2) films were analyzed by employing Lorentzian–Gaussian functions, as illustrated in Figure 3d. Clearly, as the concentration of La^3+^ increases, there is a corresponding decrease in the proportion of oxygen vacancies and an increase in the proportion of lattice oxygen. The obtained results further validate the effectiveness of introducing La^3+^ to reduce the concentration of oxygen vacancies, which is highly beneficial for improving *E*_b_.

**Figure 4:**
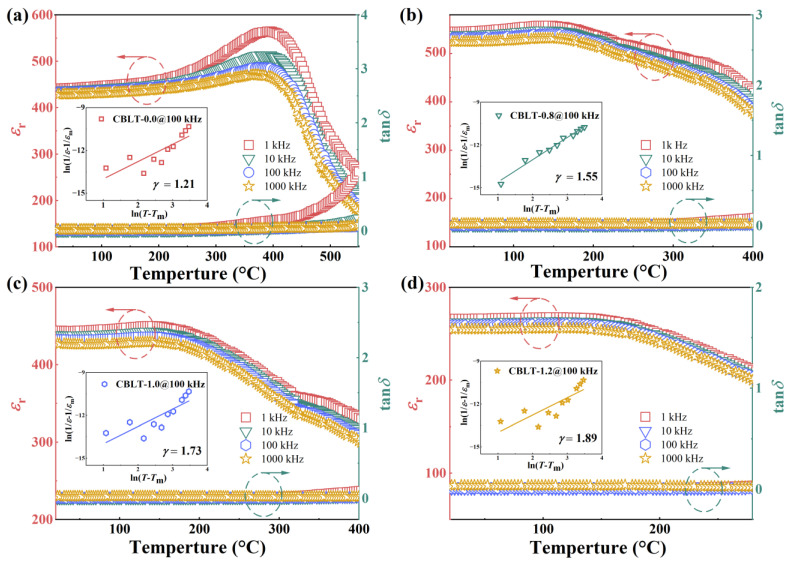
Temperature-dependent *ε*_r_ and tan*δ* measured at different frequencies of (**a**) CBLT-0.0, (**b**) CBLT-0.8, (**c**) CBLT-1.0, and (**d**) CBLT-1.2 films. The inset displays fitted curves that correspond to the modified Curie–Weiss law.

The relaxor characteristics of CBLT-*x* (*x* = 0.0, 0.8, 1.0, and 1.2) films can be determined by analyzing the temperature dependent *ε*_r_ and tan*δ*. Figure 4a–d display the *ε*_r_ and tan*δ* of CBLT-0.0, CBLT-0.8, CBLT-1.00, and CBLT-1.2 films at different measurement frequencies (1 kHz, 10 kHz, 100 kHz, and 1000 kHz) as a function of temperature, respectively. The CBLT-0.0 film exhibits a noticeable dielectric peak, reaching its maximum dielectric constant (*ε*_m_) at a temperature of 400 °C, as illustrated in Figure 4a. As the frequency increases, the peak value remains almost constant, which is considered a typical characteristic of ferroelectric materials. In contrast, CBLT-0.8, CBLT-1.00, and CBLT-1.2 films exhibit a distinct frequency dispersion phenomenon in which the peak value of *ε*_m_ moved towards the high-temperature region as the frequency increased. This behavior is considered a characteristic of RFEs. Additionally, the *T*_m_ peak of *ε*_m_ shifts towards lower temperatures as the concentration of La^3+^ increases, resulting in a broader and more diffuse distribution of peaks around *T*_m_. Simultaneously, there is a gradual decrease in the *ε*_m_, which can be attributed to weakened dipole–dipole interactions [41]. The modified Curie–Weiss law can be used to further evaluate the relaxor characteristics of CBLT-*x* films [42,43].

(4) $$\frac{1}{\varepsilon_{r}}-\frac{1}{\varepsilon_{m}}=\frac{{\left(T-T_{m}\right)}^{\gamma }}{C}$$

where *γ* represents the degree of diffusion, and *C* represents the Curie constant. The range of *γ* values from 1 to 2 signifies the transition from a conventional ferroelectric material to an ideal relaxor ferroelectric. The fitted curves corresponding to the modified Curie–Weiss law are shown in the inset of Figure 4a–d. The *γ* value of the CBLT-0.0 film is close to 1, indicating that it is a ferroelectric material. As the concentration of La^3+^ increases, the *γ* value gradually increases. The *γ* values of CBLT-1.0 and CBLT-0.0 films are approximately 2, indicating that they belong to RFEs. The strength of the Bi-O bonds is considerably higher in comparison to that of the La-O bonds. Therefore, the substitution of Bi^3+^ with La^3+^ induces a weakly coupled relaxor of the pseudo-perovskite (CaBi_2_Ti_4_O_13_)^2−^ layer in the CBLT-*x* (*x* = 0.8, 1.0, and 1.2) films. This reduction in dipole–dipole interactions between neighboring TiO_6_ octahedra disrupts the long-range ordered ferroelectric domains, leading to the formation of polar nanoregions (PNRs) [43]. Furthermore, in Figure 4a–d, the tan*δ* values of the CBLT-0.0, CBLT-0.8, CBLT-1.0, and CBLT-1.2 films were determined at 1 kHz and ambient temperatures were 0.9410, 0.1075, 0.1033, and 0.0257, respectively. It is believed that the decrease in the tan*δ* value with the increasing La^3+^ doping concentration results from a lower oxygen vacancy concentration.

**Figure 5:**
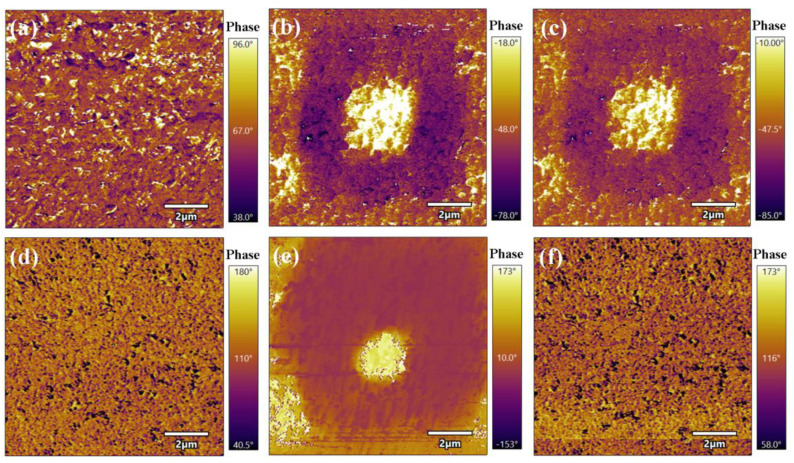
Phase PFM images, the evolution behavior of domains under ± 50 V and after a duration of 15 min for (**a**–**c**) CBLT-0.0 film and (**d**–**f**) CBLT-1.0 film.

To further confirm the relaxor behavior induced by La^3+^ doping, Figure 5 illustrates the domain structure and dynamic response of CBLT-0.0 and CBLT-1.0 films. The electrical domain structures of CBLT-0.0 and CBLT-1.0 films are exhibited in Figure 5a,d. Clearly, the CBLT-0.0 film exhibits a macroscopically long-range ordered ferroelectric domain structure with well-defined domains separated by domain boundaries, indicating its excellent ferroelectric properties. In contrast, the domain structure disorder of the CBLT-1.0 film increases, making it difficult to observe continuous large-area ferroelectric domains. The substitution of Bi^3+^ with La^3+^ weakens dipole interactions, leading to the formation of polar nanoregions (PNRs). This indicates a transition from ferroelectrics (FEs) to relaxor ferroelectrics (RFEs). The evaluation of the retention ability and dynamic performance of CBLT-0.0 and CBLT-1.0 films involved applying voltage signals ranging from +50 V to −50 V within a 2 μm range, as depicted in Figure 5b,e. It can be observed that most domains of CBLT-0.0 and CBLT-1.0 films can switch at a voltage of ±50 V. In the CBLT-0.0 film, there is a strong interaction among the long-range ordered ferroelectric domains, resulting in only a small fraction of the domains recovering to their original state within 15 min (Figure 5c). This reflects the excellent ferroelectric properties of the CBLT-0.0 film. However, almost all domains were restored to their original state within 15 min (Figure 5f), which can be attributed to the existence of high dynamic and sensitive PNRs in the CBLT-1.0 film. In general, the presence of PNRs has a positive impact on minimizing hysteresis and *P*_r_ values.

**Figure 6:**
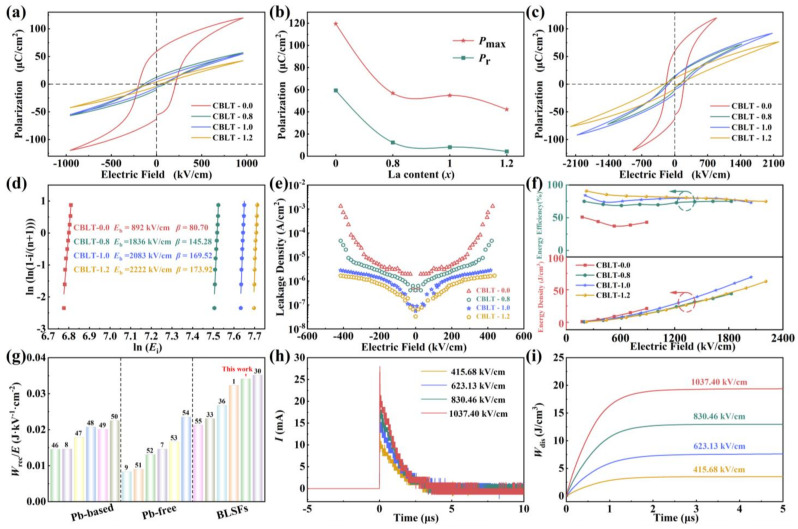
(**a**) *P-E* loops of CBLT-*x* (*x* = 0.0, 0.8, 1.0, and 1.2) films at 1040 kV/cm. (**b**) variations in the *P*_max_ and *P*_r_ values of CBLT-*x* (*x* = 0.0, 0.8, 1.0, and 1.2) films at 1040 kV/cm. (**c**) *P-E* loops of CBLT-*x* (*x* = 0.0, 0.8, 1.0, and 1.2) films were measured under *E*_b_. (**d**) The Weibull distribution of *E*_b_ for CBLT-*x* (*x* = 0.0, 0.8, 1.0, and 1.2) films. (**e**) *J–E* curves of leakage current for CBLT-*x* (*x* = 0.0, 0.8, 1.0, and 1.2) films. (**f**) the *W*_rec_ and *ƞ* of CBLT-*x* (*x* = 0.0, 0.8, 1.0, and 1.2) films. (**g**) *W*_rec_/*E* comparison of CBLT-1.0 film with other energy storage film systems. (**h**) The discharge current curve of CBLT-1.0 film under ambient conditions. (**i**) The *W*_dis_ of CBLT-1.0 film at different electric fields.

The *P-E* loops of CBLT-*x* (*x* = 0.0, 0.8, 1.0, and 1.2) films at 1040 kV/cm are displayed in Figure 6a. Obviously, the CBLT-0.0 film demonstrates a full *P-E* loop, indicating its excellent ferroelectric behavior. However, as the concentration of La^3+^ doping increases, the shape of the *P-E* loop gradually becomes narrower. This phenomenon can be attributed to the introduction of weakly coupled relaxor caused by replacing Bi^3+^ with La^3+^, Consequently, there is a transition in CBLT-*x* (*x* = 0.0, 0.8, 1.0, and 1.2) films from FEs to RFEs. The impact of La^3+^ doping concentration on *P*_r_ and *P*_max_ at a constant electric field of 1040 kV/cm is shown in Figure 6b. The *P*_max_ value gradually decreases from 119.5 μC/cm^2^ for the CBLT-0.0 film to 42.2 μC/cm^2^ for the CBLT-1.2 film. This indicates that the introduction of high La^3+^ content can suppress *P*_max_ due to its ability to enhance structural symmetry. Similarly, the *P*_r_ of CBLT-*x* (*x* = 0.0, 0.8, 1.0, and 1.2) films also exhibited a decreasing trend at 1040 kV/cm. The *P*_r_ value decreased from 59.3 μC/cm^2^ (CBLT-0.0 film) to 8.1 μC/cm^2^ (CBLT-1.0 film), then further decreased to 4.2 μC/cm^2^ (CBLT-1.2 film). This decrease in the *P*_r_ value is attributed to weak coupling relaxor. The *P-E* loops of the CBLT-*x* (*x* = 0.0, 0.8, 1.0, and 1.2) films at their respective breakdown electric fields are depicted in Figure 6c. It is evident that the *P-E* loop of the CBLT-0.0 film appears relatively full, characterized by significantly high values of *P*_max_ and *P*_r_. However, the CBLT-0.0, CBLT-0.8, CBLT-1.0, and CBLT-1.2 films show narrower *P-E* loops with gradually decreasing *P*_max_ and *P*_r_ at the maximum electric field. The *P*_max_ and *P*_r_ values observed for the CBLT-0.0, CBLT-0.8, CBLT-1.00, and CBLT-1.2 films were found to be 119.51 μC/cm^2^ and 59.3 μC/cm^2^, 56.85 μC/cm^2^ and 12.3 μC/cm^2^, and 54.81 μC/cm^2^ and 8.1 μC/cm^2^, as well as 42.2 μC/cm^2^ and 4.2 μC/cm^2^, at electric field strengths of approximately 950 kV/cm, 1400 kV/cm, 2050 kV/cm, and 2200 kV/cm, respectively. The introduction of weakly coupled relaxor disrupts the coupling of ferroelectric domains, leading to a reduction in ferroelectric properties and an enhancement of relaxor behavior. Consequently, this results in a simultaneous decrease in both *P*_max_ and *P*_r_. Additionally, with the rise in La^3+^ doping concentration, the breakdown electric field (*E*_b_) value progressively increases. This is attributed to a reduction in oxygen vacancy concentration and the inhibition of grain growth. The *E*_b_ values of the CBLT-*x* (*x* = 0.0, 0.8, 1.0, and 1.2) films were assessed using the Weibull distribution function [44]:

(5) $$P_{i}=1-\exp\left[-{\left(\frac{E}{E_{b}}\right)}^{\beta }\right]$$

where *E* and *E*_b_ denote the measured and characteristic breakdown strengths, respectively. The *E* distribution is evaluated using the shape parameter *β*, while *P*_i_ denotes the likelihood of cumulative failure. When the value of *P*_i_ is equal to or greater than 63.2%, the material will collapse. The *E*_b_ values for CBLT-*x* (*x* = 0.0, 0.8, 1.0, and 1.2) films can be obtained by linearly fitting ln[-ln(1 − *P*_i_)] and ln*E_i_*. The value of *P*_i_ is determined by the formula 1/(*n* + 1), where *n* represents the sample number, and i denotes the label number for each individual sample. As depicted in Figure 6d, the average *E*_b_ values of CBLT-0.0, CBLT-0.8, CBLT-1.0, and CBLT-1.2 films are 892 kV/cm, 1836 kV/cm, 2083 kV/cm, and 2222 kV/cm, respectively.

Obviously, with the increase of La^3+^ concentration, there was a significant enhancement observed in *E*_b_, and the *E*_b_ of the CBLT-1.2 film was 2.49 times that of the CBLT-0.0 film. The leakage current characteristic directly affects the *E*_b_ of the CBLT-*x* (*x* = 0.0, 0.8, 1.0, and 1.2) films. Figure 6e demonstrates the correlation between the applied electric field (*E*) and current density (*J*) in CBLT-*x* (*x* = 0.0, 0.8, 1.0, and 1.2) films. The leakage current densities of CBLT-0.0, CBLT-0.8, CBLT-1.0, and CBLT-1.2 films at 400 kV/cm are 1.30 × 10^−3^ A/cm^2^, 4.75 × 10^−5^ A/cm^2^, 2.78 × 10^−6^, and 1.67 × 10^−6^ A/cm^2^, respectively. The CBLT-0.0 film exhibits a large leakage current density, mainly caused by the evaporation of Bi during the high-temperature annealing process, which results in the formation of more oxygen vacancies. Increasing the La^3+^ content in the CBLT-0.8, CBLT-1.0, and CBLT-1.2 films reduces their leakage current density by suppressing the formation of oxygen vacancies and defects. Reducing the concentration of oxygen vacancies slows down grain boundary migration, thereby inhibiting grain growth. This reduction in grain size leads to an increase in the quantity of grain boundaries with high resistivity, which proves advantageous for enhancing *E*_b_ [45]. Furthermore, the reduction in leakage current density decreases the likelihood of intrinsic breakdown in the film, thereby minimizing Joule heat generation and preventing thermal breakdown. As a result, there was a significant enhancement in the *E*_b_ value of CBLT-0.8, CBLT-1.0, and CBLT-1.2 films.

The *W*_rec_ and *η* values, obtained from the *P-E* loops tested in Figure 6c, are presented in Figure 6f. The *W*_rec_ values showed a positive correlation with the electric field strength for all CBLT-*x* (*x* = 0.0, 0.8, 1.0, and 1.2) films, while an opposite trend was observed for the *η* values. The CBLT-0.0, CBLT-0.8, CBLT-1.0, and CBLT-1.2 films exhibit *W*_rec_ and *ƞ* values of 21.3 J/cm^3^ and 43%, 43.9 J/cm^3^ and 75.1%, 70 J/cm^3^ and 73%, and 62.7 J/cm^3^ and 75%, correspondingly. It is noteworthy that the *W*_rec_ value has shown a significant enhancement following the introduction of La^3+^, an observation that is quite intriguing. Among them, the CBLT-1.0 film demonstrated superior energy storage performance by exhibiting an excellent *W*_rec_ value of 70 J/cm^3^ and a significant *ƞ* value of 73% at 2050 kV/cm. The *W*_rec_ and *η* values of CBLT-1.0 film are 3.29 times and 1.4 times those of CBLT-0.0 film, respectively. The replacement of Bi^3+^ with La^3+^ disrupts the long-range ordered ferroelectric domains due to weaker orbital coupling in La-O bonds compared to Bi-O bonds. This leads to highly dynamic and sensitive polar nanoregions (PNRs), which are primarily responsible for the weakly coupled relaxor behavior. To assess the energy storage capacity of CBLT-1.0 film, its energy storage performance was compared with that of other reported film systems [1,7,8,9,30,33,36,46,47,48,49,50,51,52,53,54,55], as depicted in Figure 6g. In comparison with film systems from other publications, it can be observed that the CBLT-1.0 film exhibits a higher *W*_rec_/*E* value, indicating its capability to achieve greater energy density under low electric field conditions. This has significant practical implications for its application in energy storage devices. Therefore, the CBLT-1.0 film proves to be a highly suitable substance for capacitors used in energy storage applications. The energy storage density recoverable by the CBLT-1.0 film was evaluated through an assessment of its charging and discharging capabilities. Figure 6h demonstrates the correlation between discharge current and electric field strength (ranging from 415.68 kV/cm to 1037.40 kV/cm) for the CBLT-1.0 film. The current quickly reaches its maximum value within less than 2 μs of discharge time, showing a clear trend. The equation to determine the discharge energy density (*W*_dis_), which is influenced by the electric field, can be expressed as follows [56]:

(6) $$W_{\mathrm{dis}}=R\frac{\int I{\left(t\right)}^{2}dt}{V}$$

where *R* denotes the load resistance, and *V* represents the volume. The *W*_dis_ can be determined directly. The *W*_dis_ in Figure 6i can be derived through an integration process. The maximum current peak value (*I*_max_) shows a linear increase from approximately 10.83 mA to 21.33 mA within the electric field strengths ranging from 415.68 kV/cm to 1037.40 kV/cm, while *W*_dis_ demonstrates a parabolic rise, increasing from around 3.50 J/cm^3^ to 19.35 J/cm^3^. The discharge process of the CBLT-1.0 film is characterized by its remarkable speed, completing within a discharge duration of under 2 μs regardless of the electric fields applied. The recoverable energy storage density shows an approximate 10% difference between direct and indirect measurements, which validates the reliability of the indirect measurement data [57,58].

**Figure 7:**
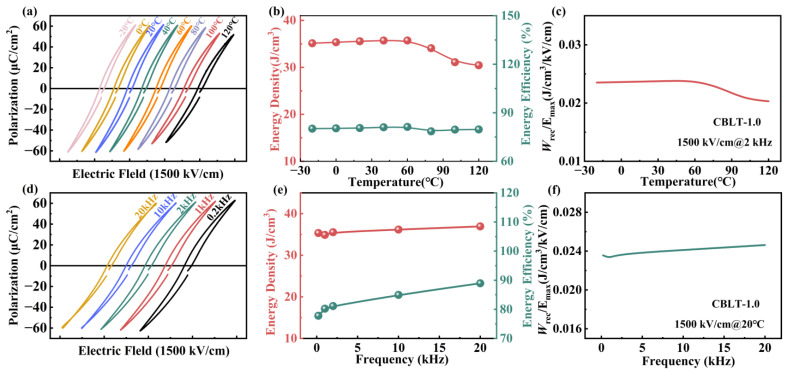
(**a**) Temperature-dependent *P-E* loops of CBLT-1.0 film at 1500 kV/cm. (**b**) The values of *W*_rec_ and *ƞ* were measured at various measured temperatures. (**c**) The variation of *W*_rec_/*E* in the CBLT-1.0 film at different temperatures. (**d**) Frequency-dependent *P-E* loops of CBLT-1.0 film at 1500 kV/cm. (**e**) The values of *W*_rec_ and *ƞ* were measured at various measured frequencies. (**f**) The variation of *W*_rec_/*E* in the CBLT-1.0 film at different frequencies.

In practical applications, the dielectric energy storage capacitor must possess excellent stability and frequency stability to ensure its normal operation under adverse conditions. The *P-E* loops of the CBLT-1.0 film measured at temperatures ranging from −20 °C to 120 °C under 1500 kV/cm are shown in Figure 7a. It can be observed that the *P-E* loops undergo slight changes as the temperature increases. The *P*_max_ value exhibits a range of fluctuations, with values ranging from 61.1 μC/cm^2^ at −20 °C to 51.9 μC/cm^2^ at 120 °C, which can be attributed to the influence of thermal variations and the progressive enhancement in domain switching polarization [59]. The energy storage performance of CBLT-1.0 film at various temperatures is illustrated in Figure 7b. As the temperature range varied from −20 °C to 120 °C, there was a decrease in both *W*_rec_ and *η* values, with *W*_rec_ dropping from 35.1 J/cm^3^ to 30.6 J/cm^3^ and *η* decreasing from 80.1% to 79.6%. This indicates that the CBLT-1.0 film exhibits good temperature stability. Figure 7c shows the variation of *W*_rec_/*E* in the CBLT-1.00 film at different temperatures. The *W*_rec_/*E* values of the CBLT-1.0 film demonstrate good temperature stability, with only a slight decrease observed over a range of −20 °C to 120 °C. Figure 7d illustrates the *P-E* loops of the CBLT-1.0 film within a frequency range spanning from 0.2 kHz to 20 kHz and an electric field strength of 1500 kV/cm. As the frequency increases, the changes in the *P-E* loops are slight. This is due to domain switching and domain wall movement lagging behind as the frequency increases, resulting in gradual increases of both *P*_max_ and *P*_r_ [60]. The *W*_rec_ and *ƞ* values of the CBLT-1.0 film at different frequencies (0.2–20 kHz) are shown in Figure 7e. The *W*_rec_ value increased slightly from 35.3 J/cm^3^ to 36.9 J/cm^3^, while the *ƞ* value increased from 77.7% to 88.9%. These findings demonstrate that the CBLT-1.0 film maintains good frequency stability. The variation of *W*_rec_/*E* in the CBLT-1.0 film at different frequencies is shown in Figure 7f. The *W*_rec_/*E* values of the CBLT-1.0 film exhibit good frequency stability, with a slight increase observed across a range from 0.2 kHz to 20 kHz.

## 4. Conclusions

To summarize, Aurivillius-phase CBLT-*x* (*x* = 0.0, 0.8, 1.0, and 1.2) films were synthesized using the sol–gel method. By replacing Bi^3+^ with La^3+^, the films incorporate weakly coupled relaxor that disrupt long-range ordered ferroelectric domains and enhance the *E*_b_. As the concentration of La^3+^ increases, the CBLT-*x* (*x* = 0.0, 0.8, 1.0, and 1.2) films demonstrate more pronounced narrowing *P-E* loops. The optimal CBLT-1.0 film demonstrates an excellent *W*_rec_ of 70 J/cm^3^ and a high *ƞ* of 73% at 2050 kV/cm. Its *W*_rec_/*E* is significantly better than most film systems from other publications. Additionally, the CBLT-1.0 film shows good thermal stability between −20 °C and 120 °C and maintains frequency stability from 0.2 kHz to 20 kHz. These results indicate that the CBLT-1.0 film exhibits great potential for utilization in energy storage purposes, and this work presents a successful approach to improving the energy storage performance of BLSF films through the formation of a weakly coupled relaxor.

## Data Availability

The original contributions presented in this study are included in the article.
